# Building Skills and Resources for Genomics, Epigenetics, and Bioinformatics Research for Africa: Report of the Joint 11th Conference of the African Society of Human Genetics and 12th H3Africa Consortium, 2018

**DOI:** 10.4269/ajtmh.19-0837

**Published:** 2020-03-23

**Authors:** Clarisse Musanabaganwa, Bonaventure Mihigo, Robert Tumusime, Mediatrice Uwanyirigira, Jorge da Rocha, Mahtaab Hayat, Melanie Govender, Peace Buto, Tina Nyunga, Raj S. Ramesar, Charles Rotimi, Jacob Souopgui, Ambroise Wonkam, Scott M. Williams, Stefan Jansen, Michèle Ramsay, Leon Mutesa

**Affiliations:** 1 Centre for Human Genetics, College of Medicine and Health Sciences, University of Rwanda, Kigali, Rwanda;; 2 Sydney Brenner Institute for Molecular Bioscience and Division of Human Genetics, Faculty of Health Sciences, University of the Witwatersrand, Johannesburg, South Africa;; 3 Division of Human Genetics, Department of Pathology, Faculty of Health Sciences, Institute of Infectious Diseases and Molecular Medicine, University of Cape Town, Cape Town, South Africa;; 4 National Human Genome Research Institute, National Institute of Health, Bethesda, Maryland;; 5 Laboratory of Embryology and Biotechnology DBM-IBMM, Université Libre de Bruxelles, Bruxelles, Belgium;; 6 Department of Epidemiology and Biostatistics, Institute of Computational Biology, Case Western Reserve University, Cleveland, Ohio;; 7 Center for Mental Health, Directorate of Research and Innovation, College of Medicine and Health Sciences, University of Rwanda, Kigali, Rwanda

## Abstract

The 11th Congress of the African Society of Human Genetics (AfSHG) was held from September 16, 2018 to September 21, 2018, in conjunction with the 12th Human Heredity and Health in Africa (H3Africa) Consortium meeting in Kigali, Rwanda. The event was organized by the AfSHG in partnership with the Rwanda Society of Human Genetics and the University of Rwanda. A 2-day workshop on the application of next-generation sequencing technologies for analyzing monogenic disease in African populations was organized as part of the conference (September 22, 2018−September 23, 2018, Kigali, Rwanda). The theme of the conference was “Building skills and resources for genomics, epigenetics and bioinformatics research for Africa.” The conference served as a platform to bring together members from country-specific Societies of Human Genetics, including Rwanda, Cameroon, Democratic Republic of Congo, Egypt, Mali, Senegal, and South Africa, and included 435 delegates from 38 countries, including 29 African countries that attended the conference. A major topic of discussion was how to bridge the gap between the emerging knowledge on genomics and Omics in African populations. The importance of understanding the role of genetic variation in disease causation and susceptibility among Africans was a constant theme during the meeting, as was the need to develop research infrastructure and resources to enhance healthcare systems, so that they are not left behind in the genomic revolution. It was concluded that there is a need to inspire more African scientists to train and work as investigators, clinicians, and genetic counselors in the field of human genetics in Africa. Local investments, and South–South and South–North collaboration were identified as the key drivers for the successful implementation of research and development on the continent.

## INTRODUCTION

**Figure 1.**
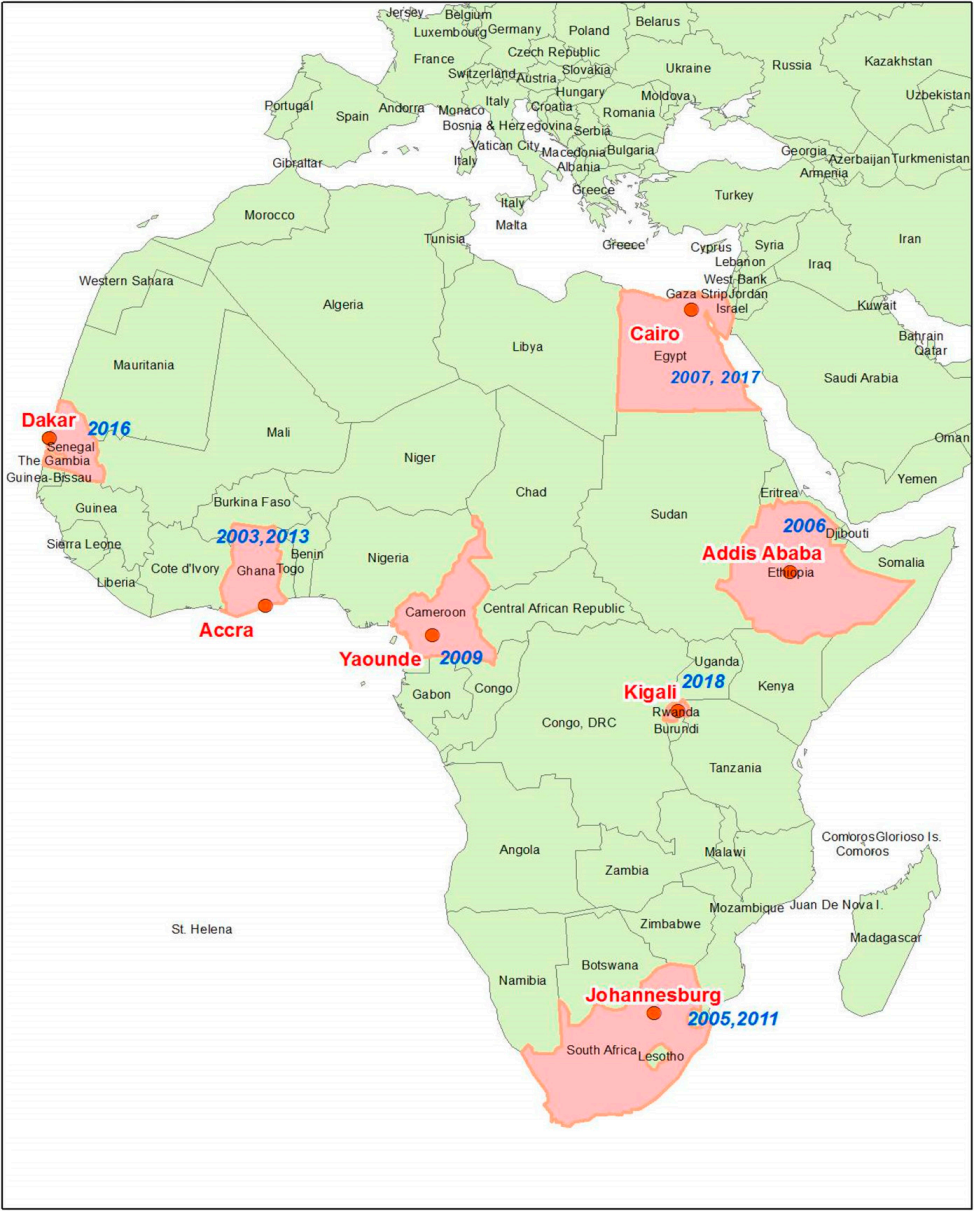
African countries that hosted African Society of Human Genetics conferences from 2003 to 2018. This figure appears in color at www.ajtmh.org.

The African Society of Human Genetics (AfSHG) in partnership with the Human Heredity and Health in Africa (H3Africa) Consortium, the Rwanda Society of Human Genetics, and University of Rwanda in Kigali organized the 11th AfSHG congress jointly with the 12th H3Africa meeting. This was the fourth collaboration between H3Africa and the AfSHG in hosting a joint conference on genomic research in Africa, following Accra (Ghana) in 2013, Dakar (Senegal) in 2016, and Cairo (Egypt) in 2017.^1^ The themes of previous AfSHG meetings that started in 2003 were as follows: “Biomedical Research in Africa with Emphasis on Genetics, *Ghana 2003*,” “Human Genetic Variation in Africa, *South Africa 2005*,” “Human Genetic Variation and Disease, *Ethiopia 2006*,” “Genomics Research in Africa: Implications for Disease Diagnosis, Treatment and Drug Development, *Egypt 2007*,” “Human Origin, Genetic Diversity and Health, *Cameroon 2009*,” “Building Capacity for Genomic and Translational Research in Africa, *South Africa 2011*,” “Advancing Genomic Research in Africa: A Joint Conference of the AfSHG and H3Africa Consortium, *Ghana 2013*,” “Strengthening Human Genetics Research in Africa, *Senegal 2016*,” and “Human Genetics and Genomics in Africa, *Egypt 2017*”^1,2^ (Figure 1).

The theme for this meeting was “Building skills and resources for genomics, epigenetics and bioinformatics research for Africa.” Leon Mutesa, chair of the local organizing committee, welcomed the delegates from different countries around the world. Michèle Ramsay, president of the AfSHG, welcomed participants and acknowledged the work carried out to prepare for the conference by the local organizers and, particularly, the young students who are the future of human genetics. She paid tribute to Bongani Mayosi and Luca Cavalli-Sforza, both recently deceased, for their contributions to human genetics over many decades. Nicola Mulder, the chair of H3Africa Steering Committee, displayed the work and contribution of H3Africa in the development of genomics in Africa. She shared different programs and opportunities for young researchers in the field (www.h3africa.org). On behalf of the government of Rwanda, the Minister of Health, Dr. Diane Gashumba, urged that critical innovations in the field of genomics are needed to reduce the burden already placed on the healthcare structures in Africa and that investment into genomics resources could help reduce this burden. The keynote speakers of the conference were Eric Green, director of the National Human Genome Research Institute of the NIH, and Solomon Ofori-Acquah, dean of the School of Biomedical and Allied Health Sciences at the University of Ghana (Table 1).

**Table 1:** Speakers of 11th conference of the African Society of Human Genetics and 12th H3Africa Consortium

Speakers of the conference	Country	Institution	Area of research
Dr. Eric Green	United States	NIH	Genomics and genetics
Dr. Solomon Ofori-Acquah	Ghana and United States	Center for Translational and International Hematology at the University of Pittsburgh	Genetic and epigenetic
Dr. Neil Hanchard	United States	Baylor College of Medicine in Houston	Molecular and human genetics
Prof. Lukusa Tshilobo	Congo	Chairs the Congolese Society of Human Genetics	Genetics
Dr. Lucien Koulischer	Belgium	University of Liège and Namur	Genetics
Dr. Nicola Mulder	South Africa	H3ABioNet	Bioinformatics
J. D. R.	South Africa	H3ABioNet	Bioinformatics
Javan Okendo	South Africa	H3ABioNet	Bioinformatics
M. R.	South Africa	H3Africa	Genetics
L. M. and Jeanne Uyisenga	Rwanda	University of Rwanda	Genetics
Guida Landoure	Mali	University of Science, Technique, and Technology of Bamako	Genetics
Christian Happi	United States/Nigeria	World Bank–funded African Center of Excellence for Genomics of infectious Diseases in Redeemer’s University	Molecular biology and genomics
Jantina de Vries	South Africa	Department of Medicine University of Cape Town	Bioethics

H3Africa = Human Heredity and Health in Africa.

### Keynote addresses.

Eric Green shared insights about the Human Genome Project and framed it as just the first step leading to more research opportunities and highlighted the phenomenal progress that has been made by AfSHG and H3Africa since 2012. He mentioned that the field of genomics is just three decades young and that we are now entering the era of genomic medicine. Tempering unrealistic expectations, he compared the developments in this field with running a marathon. He added that the field has moved from singular analyses, involving few samples, phenotypes and genes, to large-scale analyses, with multiple complex phenotypes which are being assessed longitudinally. It was noted that the cost of sequencing had plummeted, and that mobile sequencing technology is now available, for example, with the development of small devices such as the nanopore technology. With the sequencing potential today, we are increasing our understanding of the causes of rare diseases and cancer among other conditions. The priority is to focus on the future and create goals for the next 10 years and beyond. He requested input into “Genomics 2020,” an initiative that aims to get suggestions and advice from researchers around the world about how National Human Genome Research Institute can help shape the future of genomics.

Solomon Ofori-Acquah shared his research on the genetic and epigenetic modulation of acute chest syndrome (ACS) in sickle cell disease (SCD). His study was conducted on 942 African patients with ACS, a condition that leads to premature death in SCD. Thirty percent of SCD patients experience ACS. He concluded by highlighting that the symptoms of ASC are decreased in individuals with specific genetic variants and proposed that genetic and epigenetic modulators of ACS could be attractive therapeutic targets in SCD.

**Figure 2.**
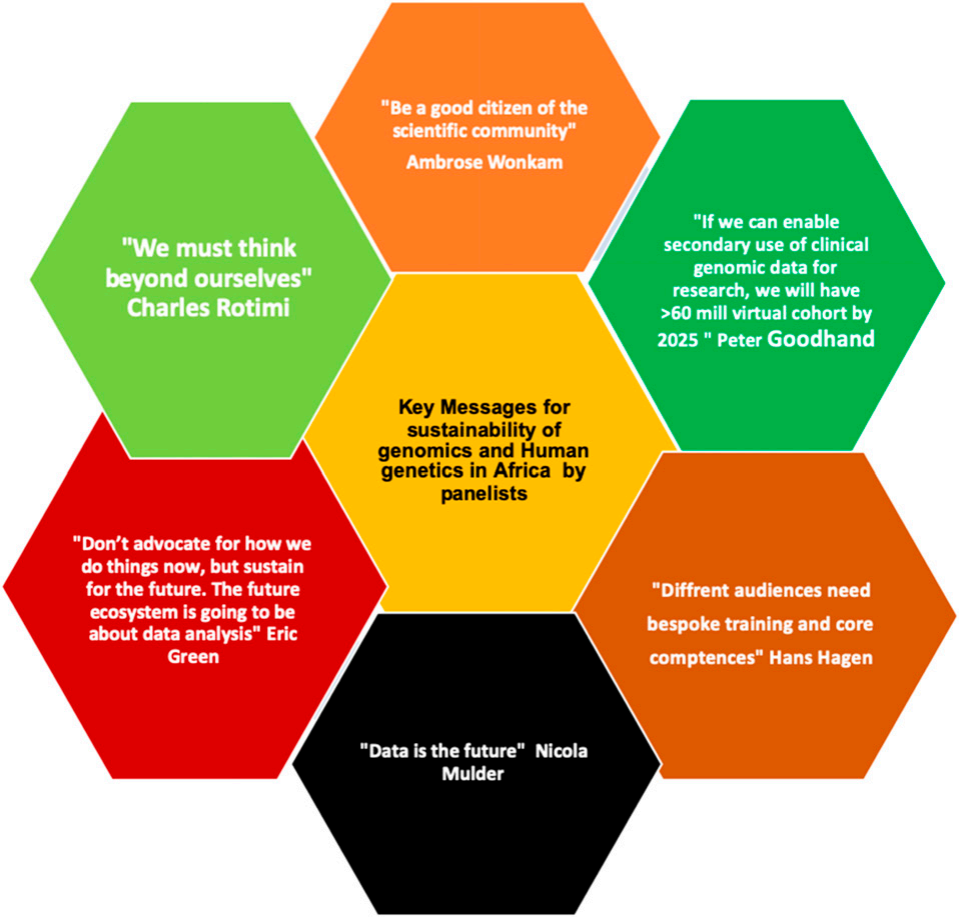
Quotes by panelists on Sustainability of Genomics and Human Genetics in Africa. This figure appears in color at www.ajtmh.org.

Three panel discussions were held focusing on the “Sustainability of Genomics and Human Genetics in Africa,” “Translational Genomics,” and “Strategic planning for the AfSHG: Building skills and resources for genomics, epigenetics and bioinformatics research for Africa” (Figure 2).

## SCIENTIFIC HIGHLIGHTS OF THE CONGRESS

### African population genetics.

Neil Hanchard presented the results of a population genetics article entitled “Diverse African Populations Inform Migration History and Disease-Mapping in sub-Saharan Africa” on behalf of the H3Africa Genome Analysis working group. He showed how novel whole genome sequences of more than 400 African individuals of 13 ethnolinguistic groups provided new understandings about migration patterns and admixture across sub-Saharan Africa over the past 4,000–5,000 years. Sequencing from African populations continues to be a source of novel variant discovery and about 3.4 million new single-nucleotide variants (SNVs) were detected, although most of them are at low frequency. He hypothesized that common loss of function alleles, if shared between groups, may have significant effects on human diseases. He urged that researchers could prioritize studies on genetic variation associated with the immune system, cancer, and cardiometabolic disorders and correlates with patterns of mortality.

Lucien Koulischer is among the first international doctors who came into Rwanda after the 1994 genocide against the Tutsi population. Besides, he is among the instrumental doctors in the journey of reestablishing the Faculty of Medicine at the University of Rwanda. He gave an eloquent address providing different types of evidence for the “Out of Africa” hypothesis. He informed the audience about the emergence of humankind, with the genus *Homo* appearing two million years ago, and how all members of the genus disappeared, with the exception of *Homo sapiens.* Referring to the Neanderthals who lived until ∼40 thousand years ago, he explained how the study of ancient genetics has led to the birth of the new field that is referred to as archaeogenetic or paleo genomics. In addition, he highlighted that in Europe, which has a colder climate than Africa, DNA is generally better preserved for such studies, but still, a few older African specimens are being discovered that were used for ancient DNA research. The first migration of *Homo sapien*s from the African continent is thought to have occurred ∼150 thousand years ago, with additional waves of migration, including the recent migration to the Americas only 14,000–20,000 years ago when the first Homo sapiens cross the Bering Sea from Siberia to America. Once there, they migrated south, populating North and Central America. Staring from the fact that it is impossible to know why they migrated, he made a compelling argument that our species will always find ways to migrate.

### Bioinformatics training and young researchers.

Human resource capabilities to analyze and interpret genomic data are considered a crucial component of modern biomedical research.^3^ Therefore, the congress included a session on the strategies to advance human resource capacity in the field of Bioinformatics. Nicola Mulder, leader of the Pan-African Bioinformatics network, H3ABioNet, gave the first presentation sharing experiences in building bioinformatics skills in Africa. She described several programs and resources offered by H3ABioNet and shared the journey from the foundation of H3ABioNet to developing bioinformatics and computational support for researchers across Africa, specifically targeting those engaged with H3Africa projects. The different training approaches were described, each with their own strengths and weaknesses. Although face-to-face workshops and courses had the highest proportion of attendees who returned positive feedback, such an approach could only support a limited number of attendees and was expensive to run. Distance-based learning methods on the other hand, such as the Introduction to Bioinformatics course, were massively successful in reaching a large number of attendees across the continent. This method uses a combination of local classrooms and prerecorded lectures to host sessions continent-wide, while providing a real-time chat forum and link between all classrooms and to the instructor. These were limited in the ability to provide specific skills needed for specific research areas but were largely successful in reaching many Africans at a low cost.

Next, several young scientists shared their findings. Variability in key pharmacogenes (*CYP2D6* and *DPYD*) was shown to be significantly different between African populations, indicating the need to develop accurate treatment guidelines for Africans. Jorge da Rocha shared the findings from his MSc research study that focused on pharmacogenomically relevant variants in genes involved in the metabolism of cancer drugs. He annotated genes with known drug reactions and used public whole-genome sequence data to identify and rank variants according to predicted function. He then examined their frequency across global populations, identifying those at high frequency in Africans. Joel Defo presented his research on genetic dating and patterns of admixture in modern human evolution, using admixture dating through simulations. He concluded by highlighting that available tools are limited in accurately determining admixture. Jeanne Uyisenga discussed the findings of the study entitled “Germline mutations in young Rwandan women with breast cancers,” which included the targeted sequencing of *BRCA1* and *BRCA2* to reveal known and novel mutations. In summary, the session concluded with a renewed awareness that the vast genomic diversity in Africans is likely to provide not only new challenges but also new opportunities for young African researchers.

### Human Heredity and Health in Africa consortium showcase.

A session was dedicated to showcasing the H3Arica projects and included talks on monogenic neurological traits, complex traits, infectious diseases, and ethical issues and concluded with a presentation on the African Academy of Science (AAS) Open Research Platform.

Guida Landoure from Mali referred to Africa as a region of opportunity for genetic studies, highlighting that Africa today is not at the forefront of this field of research. He attributes this to a lack of resources, more specifically human resources (weakened by the brain drain) and a lack of training opportunities. Furthermore, he said that many research projects carried out on African populations and the African diaspora were performed by non-Africans, and publications often had no authors from the continent. This is partly due to limited research funding from local governments. There is, however, positive news as a greater awareness of fair attribution and avoidance of stigmatization lead to greater engagement with African scientists. Among others, breakthroughs are being made in discovering the genetic mutations, often novel mutations, that cause rare neurological diseases in African families.

Michèle Ramsay presented the work of an H3Africa Collaborative Center referred to as AWI-Gen (Africa Wits-INDEPTH partnership for genomic studies) where they recruited more than 12,000 adult participants in a cross-sectional population study to identify genetic and environmental contributions to obesity and cardiometabolic diseases. Their study showed that obesity is common in East and South African and women are disproportionally burdened. Often as a consequence of obesity, hypertension and diabetes were also common. Within rural West African communities, there was less obesity, although hypertension was present in about 20% of the participants. Preliminary genome-wide analyses using the H3Africa SNVs genotyping array showed some replication of previous association signals with body mass index.

Christian Happi spoke on genomic surveillance and characterization of microbial threats in West Africa. He referred to his work during the 2014 Ebola outbreak and the fact that they immediately made publicly available the sequences of 99 Ebola genomes, and later a further 150 genomes from Nigeria and Sierra Leone, to ensure that they could accelerate research and the development of diagnostic tests. The Ebola viral genomes revealed properties related to the spread of the epidemic. They created a consortium building on long-standing research training partnerships to do microbial metagenomics for viral pathogens, develop field diagnostics for pathogen surveillance, and use the clinical genomic and immunological data to study viral and host biology. The main aim of the consortium is to build research capacity in genomics, use genomic bedside tools to control and eliminate infectious diseases, innovate on genomics curricula, and engage communities. Three groups focusing on education, genomics capacity building, and research aim to develop a genomic-based diagnostic service based on targeted sequencing.

Jantina de Vries shared highlights from the ethics working group of the H3Africa Consortium and the guidelines on community engagement, informed consent, and biobanking in an African context. The aim was to make the document easily available to empower researchers working in the field of genomics in Africa and to inform ethics review committees. Through the consortium, African bioethicists are being trained, but there is a need to build a critical mass of bioethics scholars and bioethics leadership to grow the evidence base for genomics research in Africa, to continue engagement and training of research ethics committees, and to develop implementation guidelines. The ecosystem of genomic research and biobanking in Africa is developing as a way to allow ethical practices to spread, promoting an accumulation of knowledge to underpin precision medicine in Africa.

The last presentation of the session was by Elizabeth Marincola, creating awareness of the AAS Open Research Publishing Platform. She shared ideas to promote a transparent, open-access research publishing ethos that would lead to greater access to state-of-the-art literature. Standards of excellence will be enhanced by an open and fully transparent review process, and articles will be listed in PubMed and Google Scholar. The downside is that authors will need to pay amounts that are often unaffordable to African researchers to publish their work. The AfSHG has initiated a discussion to have a dedicated society portal on the AAS Open Research Platform and is working with the AAS and F1000 (who hosts the platform) to provide sponsorships to allow some African researchers to publish at nominal cost.

### Epigenomics and functional genomics.

Epigenomics is the field of study that looks at the functional regulating of genes and is seen as a new approach to understanding common disease.^4^ The first presentation by Monica Uddin talked about a study that aims to explore intergenerational transmission of epigenetic modulation and gene expression related to exposure to 1994 genocide against the Tutsi population and their offspring. In the presentation, she highlighted that the heritability of post-traumatic stress disorders (PTSD) is estimated at 30% leaving 70% of variability in the trait to be explained by environmental factors.^5^ She suggested that if there was a causal epigenetic mechanism, this could provide an opportunity to alter or regulate gene expression, without modifying the DNA, which can lead to improved management and perhaps prevention of PTSD. The epigenetic mechanism of PTSD could include changes in DNA methylation and preliminary studies showed the associations with DNA methylation. Salah Eddine Ayoubi shared his findings on gene expression profiling for breast cancer in Moroccan women and suggested that research findings may lead to potential novel therapeutic targets. Amadou Gaye discussed transcriptome signatures for hypertension in African Americans using a machine learning approach, and Paule Joseph spoke about the role of traumatic life events on miRNA expression in differential pain profiles.

### Genomics of rare diseases.

The session was opened by Youssef Idaghdour, with insights into novel ways to investigate malaria, including characterization of in vivo host response to falciparum infection. Gene expression is useful to characterize genetic disease, as it assists with providing more power to detect changes related to disease susceptibility and progression. He highlighted the use of studying miRNA, which shows large variability in expression throughout infection providing substantially more power to detect associations with parasitemia, which other genetic markers (such as SNPs) cannot provide.

Other talks in this session drew attention to the importance of characterizing rare diseases in Africa. Congenital malformations were discussed as well as their sociocultural impact on African families. These disorders are not well understood by the public, which has led to family disrupting misconceptions. A research group led by J. S. at the Free University of Brussels assessed holoprosencephaly, a disease which is currently not well understood in terms of molecular etiology. Using animal models, they found that signaling pathways involving the Spemann’s Organizer are perturbed in patients presenting with holocephaly. This work is being carried out on the continent and has been reported to have large implications for education among communities, which may lessen the burden to families already facing such diseases. Aime Lumaka presented work highlighting challenges encountered when using next-generation sequencing (NGS) to investigate rare diseases in Africa. In an assessment of Congolese and Belgian patients with rare diseases, it was clear that diversity in African genomes is difficult to interpret as current databases and guidelines are based on data from non-African populations. Aime Lumaka discussed the importance of rare diseases research in the Democratic Republic of Congo. He showed that rare diseases affect > 1 in 2,000, equaling to 40 individuals affected with at least one rare disease per km.^2^ Africans have different presentation of disease symptomology from Caucasians, which can be due to differences in genetic background. He presented an algorithm that recognizes facial features associated with genetic conditions.^5^

He suggested that because the existing catalogs are biased, there is a need to include more African patients, highlighting the need to study rare diseases in Africa.

Annete Uwineza closed the session presenting whole-exome sequencing (WES) data from Rwandan patients with neurodevelopmental disorders. Three Rwandan families were assessed and pathogenic variants were identified in the *PEX13* gene in a consanguineous family, with known links to biogenesis disorders. A dominant mutation in *EFTUD2* was identified in another family. This work demonstrated the ability of WES sequencing to be useful for diagnosis and characterization in Rwanda, although the authors note that strong selection criteria are necessary in selecting cases, as available funding is limited.

Zane Lombard and Ghada Kamah facilitated the second part of the genomics of rare diseases’ session on the final day of the congress.

Rokhaya Ndiaye presented on genetic diversity in patients with albinism in Senegal. In the preliminary study, they examined 10 cases with albinism by screening for mutations in 19 genes. Variants found in Senegalese patients with oculocutaneous albinism differed from those found in South Africans with albinism, and a larger study would be needed to identify founder mutations. She concluded by recommending that genetic counseling services be implemented for people with albinism and that this should include education about the increased risk for early development of skin cancer that could be prevented by using protective measures from an early age. Melanie Govender discussed genetic associations with focal segmental glomerulosclerosis (FSGS) in black South African children. She showed that apolipoprotein1 (*APOL1*) kidney risk variants were not significantly associated with FSGS in this group, and that progression to end-stage renal disease was significantly faster in black South African children with steroid-resistant nephrotic syndrome (SRNS) in the presence of the *NPHS2* V260E variant. She concluded by saying that in black South African children with FSGS, testing the *NPHS2* V260E mutation could confirm a diagnosis of SRNS, inform treatment options, prevent invasive renal biopsy, and provide an opportunity for accurate genetic counseling and informed reproductive choices for families. Mohamed Alimohamed discussed the routine use of targeted NGS of a gene panel in a Dutch cardiomyopathy cohort. He showed that cardiomyopathies are a group of diseases that can be acquired or inherited. There are different subtypes, including dilated, hypertrophic, restrictive, arrhythmogenic right ventricular, specific, and nonclassified cardiomyopathies. Targeted NGS has revolutionized the way multiple genes are simultaneously examined, and this can be evaluated against the use of WES that is more expensive. Jean Pascal Demba Diop examined the role of *BRCA1*, the gene that predisposes individuals to hereditary breast cancer, in Senegalese patients. They identified a founder mutation (c.815_824dup10), and although several studies have reported specific pathogenic variants in sub-Saharan African populations, this variant appears to be of Senegalese origin. Haplotype studies are required to estimate when the mutation first arose.

### Host genetics and infectious disease.

An emerging field of research has demonstrated the role of host and pathogen genetics in the management of infectious diseases.^6^ The session focused on the importance of considering infectious diseases in the context of disease etiology and, more specifically, their effect on the human host and its response to infection. Caroline Tiemessen discussed models for the study of HIV remission focusing on elite and posttreatment controllers. She highlighted the importance of the timing of initiation of antiretroviral therapy in relation to the time of HIV-1 acquisition that comes from adult studies with the identification of “posttreatment controllers,” and from the pediatrics remission cases of the “Mississippi baby,” the “French teenager,” and the “South African child.” Marlo Moller presented research on the role of ancestry in tuberculosis susceptibility in the South African context, especially in admixed populations from the Western Cape Province. She emphasized the importance of considering biological sex, host pathogen coevolution, and primary immunodeficiency.

Fepa Gaelle from Cameroon presented on hepatitis C virus (HCV) 1b and 4a subtypes. She mentioned that those HCV subtypes are the most virulent and, therefore, require special care. She highlighted that the HCV clones generated by their study may establish useful and representative molecular tools for further investigations regarding the life cycle, persistence of the virus, as well as vaccine and drug development. Ultimately, Olayinka Kotila from Nigeria provided insights on the N-acetyltransferase II discordant genotype and phenotype relationship in HIV-positive and HIV-negative Nigerians. In the discussion, it was highlighted that the identification of *NAT2* genotype in African populations was performed by using N-acetyltransferase. The audience learned that NAT2 haplotypes are similar in Nigerians, irrespective of HIV status. Genotypically, Nigerians are majorly slow acetylators, but phenotype data showed the difference.

### Ethics, genomic research, and translation in Africa.

Advances have been made in considering ethical issues that arise when doing genomic research in an African context, and a session was dedicated to this topic. Paulina Tindana’s presentation focused on the role of community engagement and noted that although researchers are often funded for costly biobanking activities and genomic data generation, there is seldom sufficient funding to support community engagement efforts before the research, with feedback during the study and after the research has been completed. There is, therefore, an urgent need to secure funding to engage researchers and participant communities and to develop guidelines and policies appropriate for the continent. Promoting ethical reflection on research processes will benefit knowledge generation and engagement with communities in many ways. This can include innovative approaches such as social media, designing and printing comic books with relevant messages and easily understood content, and short videos that would demystify biobanking research. Yasmin Aguib addressed the difficult question of what constitutes a clinically actionable genetic variant in the population of her country, Egypt. Ongoing studies within the H3Africa Consortium are currently grappling with this tough question and how to address it in an African context where there is still a dearth of data and knowledge of genetic links to disease causality.

Translation of genomic research findings is a challenge, as are concepts of data ownership and protection of participants. Janine Scholefield noted that drug trials are usually conducted on minimally diverse genetic populations and that there is little consideration for populations heavily burdened by HIV infection and tuberculosis. This often leads to the common occurrence of adverse drug reactions, either due to common genetic variants in genes involved in drug absorption, distribution, metabolism, and excretion (referred to as ADME genes) in African populations or because of drug–drug interactions that are present in patients with multiple morbidities. Scholefield’s research uses an in vitro cellular model where she produces induced pluripotent stem cells from skin biopsies to assess the metabolism and action of specific drugs on cells with genetic variants that are more common in Africans. Ghada El-Kamah gave an excellent and thought-provoking presentation on key issues to consider in an ethics regulatory framework for patient registries, drawing from her experiences in Egypt. She argued that handling big data is a major challenge for many academics and that it is essential to carefully manage and coordinate a registry, especially if the aim is to use it to enrich research that will be useful to the healthcare system in a country. Dealing with informed consent can be a challenge, for example, under some circumstances, informed consent could be unobtainable (e.g., deceased patient where contact has been lost with the family). It is, therefore, important to implement a prospective consent process and to assure participants of confidentiality and the efforts in place to protect their privacy and interests. Ownership of data and samples can also be a thorny issue. She asked who would decide on reasonable and ethical access to these resources and warned that unnecessary restrictions could severely hamper research and the advancement of knowledge.

### Complex disease genomics.

The common occurrence of many complex diseases, such as diabetes and hypertension, have become a major public health problem^7^ and, therefore, merited a session on complex diseases and associated genomics. Adebowale Adeyemo touched on recent advances in the genetics of nephrotic syndrome in childhood and ways to improve diagnostic access in African patients.

Dezheng Huo discussed inheritable breast cancer in Nigeria, Uganda, and Cameroon. He claimed that breast cancer is the most common cancer in Africa affecting 24% of African women. In addition, the mortality rate is high. In developed and well-resourced environments, gene expression profiling and the analysis of the whole genomes of breast cancers is becoming common, but challenges persist in Africa.

### Anatole Laleye discussed immunogenetic aspects of type 1 diabetes in Benin.

He reminded the audience that type 1 diabetes is a chronic metabolic disorder with an autoimmune etiology, characterized by the lack of insulin. He highlighted that in 2007, the prevalence of diabetes was 0.5% expecting millions of people to be having diabetes by 2025.^8^ Fatima Doumatey discussed the molecular study of genetic renal diseases in the Moroccan population and insights into gut microbiota dysbiosis in Africans with type 2 diabetes (T2D). She argued that T2D is a public health problem as Africa has the highest prevalence (at 2.6%) when compared with other continents.

## CONCLUSION

**Figure 3.**
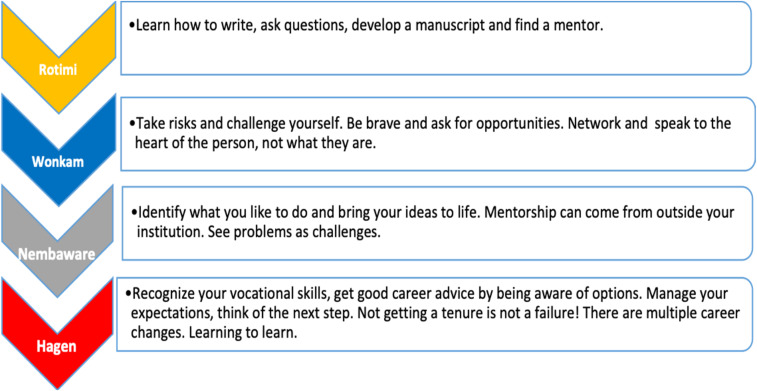
Advice on career opportunities for young African geneticists (workshop). This figure appears in color at www.ajtmh.org.

The plenary discussion on future perspectives for AfSHG and the H3Africa Consortium was the last session of the conference (Figure 3). The conference suggested that the main focus in the future should be on development of human resource capabilities for African scientists in bioinformatics, medical genetics, and genomics in Africa. While acknowledging that the completion of the Human Genome Project created whole new fields of research that African scientists should engage with, scientists showed how technological advancement has led to the reduction of sequencing cost, making it more accessible to researchers across the world. African governments should invest in genetics and genomics research to advance in medical research on the continent, and this may lead to the development of African research agenda that spotlight genetics and genomics in decision-making with in the healthcare delivery. Besides, key speakers in the conference underlined that future of human genetics and genomics in Africa should make better use of existing data and repositories while considering the protection of rights and privacy of the patient. Clinicians were encouraged to engage in future research as they have access to massive amounts of important data that may inform their decision in clinical practices. Going forward, scientists urged that a culture of data sharing on the continent may speed up the advancement of human genetics and genomics research in Africa and across the world. The “Genomics 2020” platform was recommended as an opportunity to suggest enabling tools that African scientists could tap into. Moreover, AfSHG was highlighted as the principle vehicle to bring African geneticists and genomics researchers together for medical research advancement in the African continent.

The 11th AfSHG Conference’s scientific sessions allowed sharing of research advances. Many African scientists had the opportunity to showcase their findings to an expert audience. The conference organizers announced winners for the best oral and poster presentations as one of the strategies to empower young scientists. The prizes included 10 subscriptions to the Nature Genetics, four scholarships to attend the second Winter Institute in Statistical Genetics at the New York University Abu Dhabi, and books. The next conference will be held at Bamako, Mali, from September 19, 2019 to September 21, 2019 (see announcement on www.afshg.org).
